# Evolution and genetic diversity of atypical porcine pestivirus (APPV) from piglets with congenital tremor in Guangxi Province, Southern China

**DOI:** 10.1002/vms3.407

**Published:** 2020-12-13

**Authors:** Kaichuang Shi, Shouyu Xie, Wenchao Sun, Huixin Liu, Jing Zhao, Yanwen Yin, Hongbin Si, Sujie Qu, Wenjun Lu

**Affiliations:** ^1^ College of Animal Science and Technology Guangxi University Nanning China; ^2^ Guangxi Center for Animal Disease Control and Prevention Nanning China; ^3^ Institute of Virology Wenzhou University Wenzhou China

**Keywords:** atypical porcine pestivirus, evolution, genetic diversity, genomic characterization

## Abstract

Atypical porcine pestivirus (APPV) was identified and associated with congenital tremor (CT) type A‐II in new born piglets and has been reported in many countries. In China, the first APPV identification in swine herds was reported in Guangdong province in 2016. To investigate the genetic characteristics of APPV in Guangxi province, 53 tissue samples from neonatal piglets with CT were collected and detected from October 2017 to May 2019. Five APPV strains which were named as GX04/2017, GX01‐2018, GX02‐2018, GX01‐2019 and GX02‐2019 were obtained. Sequence analysis revealed that all six APPV strains from Guangxi province, including five strains from this study and one from a previous report, shared 83.3%‐97.5% nucleotide identity of complete genome and 91.7%‐99.1% amino acid identity of the open reading frame (ORF), and shared 77.7%‐97.7% nucleotide identity of complete genome and 90.6%‐99.3% amino acid identity of ORF with reference strains. Phylogenetic analysis indicated that all APPV strains could be divided into three clades based on the complete genome, N^pro^, E^rns^ and E2 gene sequences, respectively; and the APPV strains from Guangxi province distributed in two clades (clades I and II). No sign of recombination was observed from Guangxi strains. Evolution analysis performed on the complete genome of 58 APPV strains showed that America, Europe and Asia strains during 2006–2019 evolved at a mean rate of 1.37 × 10^–4^ substitutions/site/year, and the most recent common ancestor (tMRCA) of them was estimated as 1,700.5 years ago. The findings of this study indicated that there existed a high degree of genetic diversity of APPV from Guangxi province, Southern China, which provided important information on the epidemiological features and evolutionary relationships of APPV.

## Graphical abstract

The complete genomic sequences of five APPV strains were obtained from positive clinical tissue samples from neonatal piglets with CT in Guangxi province, Southern China. The five APPV strains distributed in two clades in phylogenetic trees based on complete genome, Npro, Erns and E2 gene sequences, respectively, which indicated that APPV strains from Guangxi province existed high degree of genetic diversity and variation. The results provided valuable information on epidemiological and evolutionary characteristics of APPV in Southern China.

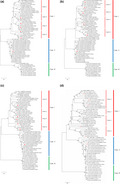

## 1 INTRODUCTION

Atypical porcine pestivirus (APPV), which is belongs to the genus *Pestivirus* of family *Flaviviridae* (Smith et al., 2017), was first discovered and identified by next‐generation sequencing (NGS) from clinical samples in the United States in 2015 (Hause et al., 2015). Since then, the presence of APPV in newborn piglets with congenital tremor (CT) has been reported in many countries, such as the United States (Arruda et al., 2016), the Netherlands (de Groof et al., 2016), Germany (Postel et al., 2016), Austria (Schwarz et al., 2017), Spain (Munoz‐Gonzalez et al., 2017), Brazil (Mosena et al., 2018), Hungary (Denes et al., 2018), England (Williamson, 2017), Canada (Dessureault et al., 2018), Sweden (Blomstrom et al., 2016), China (Zhang et al., 2017), Korea (Kim et al., 2017), Switzerland (Kaufmann et al., 2019) and Italy (Sozzi et al., 2019), and so on. However, several retrospective studies showed that APPV might has been circulating in swine herds of some countries for decades. APPV was identified in different tissues of the CT affected piglets from 1997 to 2016 in Spain (Postel et al., 2016), was identified in lymph nodes collected in 2007 from Swedish pigs suffering from postweaning multisystemic wasting syndrome (PMWS) (Blomstrom et al., 2016), and was detected in serum samples from the indigenous pig populations in Switzerland as far back as 1986 (Kaufmann et al., 2019). In China, the first APPV identification in swine herds was reported in Guangdong province in 2016 (Zhang et al., 2017), and subsequently, the genomic presences of APPV were detected in pigs from Guangxi, Guizhou, Jiangxi, Yunnan, Anhui and other provinces (Pan et al., 2019; Shen et al., 2018; Wu et al., 2018; Xie et al., 2019; Yan et al., 2019; Zhang et al., 2018; Zhou et al., 2019).

APPV has been discovered in both domestic pigs and wild boar populations (Cagatay et al., 2018; Gatto et al., 2018; Sozzi et al., 2019). The clinical presentation of APPV‐infected pigs was characterized by CT type A‐II in piglets, whereas adult pigs might become persistent carriers and shedders (de Groof et al., 2016; Schwarz et al., 2017). High APPV loads were detected by qRT‐PCR in semen, serum and different tissue samples of infected pigs (Gatto et al., 2018; Liu et al., 2019; Schwarz et al., 2017). Besides horizontal transmission through oronasal pathway, APPV could also be vertically transmitted by transplacental infection (Arruda et al., 2016; de Groof et al., 2016). It could occur as a sporadic disease affecting single litters or as an outbreak over several weeks affecting multiple litters, and the losses of 2.5 piglets per sow and a 10% drop in pig reproductive performance was occurred on a farm in Austria (Schwarz et al., 2017). One retrospective study showed that 41.8% (51/122) sampled pig farms and 16.3% (182/1115) porcine serum samples could be detected antibodies against APPV in Germany (Michelitsch et al., 2019). Due to the abovementioned complicated epidemiology and the huge loss of infected swine herds, APPV has attracted great attention in many countries.

APPV is a newly discovered virus. Two independent studies have been reported that CT was reproduced following experimental inoculation with serum or tissue‐homogenate‐pools containing APPV (Arruda et al., 2016; de Groof et al., 2016), and APPV has been proven to fulfil Mokili's Metagenomic Koch's Postulates, so APPV is associated with CT type A‐II (Stenberg et al., 2020). Some studies on APPV molecular epidemiology have found that there existed high genetic variation among different strains (Choe et al., 2020; Pan, Mou, et al., 2019; Schwarz et al., 2017; Williamson, 2017; Wu et al., 2018; Zhang et al., 2017; Zhou et al., 2019), with up to 21% genetic distance among the viruses (Postel et al., 2017). However, APPV strains available for biological, origin and evolution analysis are still scarce so far, and it is inadequate for studies on understanding APPV genetic diversity and evolutionary relationships. This study was intended to investigate the evolutionary relationships and genetic diversity of APPV strains from Guangxi province, Southern China. The results would provide valuable information for research on epidemiological and evolutionary characteristics of APPV in China.

## 2 MATERIALS AND METHODS

### 2.1 Collection of clinical samples

From October 2017 to May 2019, newborn piglets with clinical CT were reported from eighteen farms in Guangxi province. Piglets presented CT soon after birth and some of them died within a week. Fifty‐three samples, including brain, liver, spleen and lymph node from each dead or sick piglet, were collected and submitted to our laboratory for diagnostic investigation. Identification of CT‐associated viral pathogens was detected by PCR or RT‐PCR for APPV, classical swine fever virus (CSFV), porcine circovirus type 2 (PCV2), porcine pseudorabies virus (PRV) and Japanese encephalitis virus (JEV).

### 2.2 Detection and sequence determination of APPV genome

The tissue samples were homogenized in phosphate‐buffered saline solution (PBS, pH7.2) and used to determine APPV presence by RT‐PCR. Viral RNA was extracted from the tissue supernatants using MiniBEST RNA/DNA Extraction Kit (TaKaRa, Dalian, China) and reverse transcribed to cDNA using PrimeScriptⅡ1st Strand cDNA Synthesis Kit (TaKaRa, Dalian, China). Then, APPV was detected by RT‐PCR to amplify 275 bp fragment of E^rns^ gene with primers F5′‐TGGGGGAAAGGGGTTAACCAG‐3′ and R5′‐ATCCGCCGGCACTCTATCAAG‐3′ using *Taq* PCR MasterMix kit (TaKaRa, Dalian, China). The APPV‐positive samples were selected randomly for amplifying the complete genome of APPV.

To determine the complete genome, RT‐PCR was used with eight pairs of specific primers (Table 1) to amplify eight overlapping fragments encompassing the open reading frame (ORF) of APPV strains using TKs Gflex DNA Polymerase Kit (TaKaRa, Dalian, China). The 5′ untranslated region (UTR) and 3′ UTR were obtained by rapid amplification of cDNA ends (RACE) using SMARTer RACE 5′/3′ Kit (TaKaRa, Dalian, China). The amplified fragments were purified and cloned into pMD18‐T vector (TaKaRa, Dalian, China) and sequenced with an ABI 3730XL sequencer.

**Table 1:** Primers used for amplifying the complete genome of APPV

Primer	Sequence (5′→3′)	Target position	Product/bp	Template
5'RACE	5′‐CGCGGATCCACAGCCTACTGATGATCAGTCGATG‐3′	/	336	000515
5'‐Inner	5′‐CCGGCACTCTATCAAGCAGTAAGGTC‐3′	311–336		
P1‐F	5′‐GCATAATGCTTTGATTGGCTGCAT‐3′	1–24	1,432	
P1‐R	5′‐GGCTTGTRTCTATCATTCCCAG‐3′	1411–1432		
P2‐F	5′‐AAGGTYCAGTGGTTCTTAAAGG‐3′	1258–1279	2,114	
P2‐R	5′‐AGRAAGCTCAAGGCTACTGGAC‐3′	3350–3371		
P3‐F	5′‐TGTGGAAAATGGACTGGACAGA‐3′	3239–3260	1902	
P3‐R	5′‐ACCTCATRAAGCGRCAGACACT‐3′	5119–5140		
P4‐F	5′‐GAATTGGCAGATATGCGGAGG‐3′	4969–4989	1783	
P4‐R	5′‐CCTGATGYTTCCTCAAGTAYTG‐3′	6730–6751		
P5‐F	5′‐GACCAYCAACTGAGGCAACTAC‐3′	6601–6622	1627	
P5‐R	5′‐TCTTGGATCCACGRTGTGCTTT‐3′	8206–8227		
P6‐F	5′‐GCCAAGTGGCCATAGGYAAAGT‐3′	8102–8123	2022	
P6‐R	5′‐ACTGAGCCCAATCTGCACTBAC‐3′	10102–10123		
P7‐F	5′‐AGAAACCACGTGTGATACAGT‐3′	9962–9982	1,495	
P7‐R	5′‐CAAGTATTTACAACAACCCCAT‐3′	11435–11456		
P8‐F	5′‐AGAAACCACGTGTGATACAGT‐3′	9962–9982	1602	
P8‐R	5′‐TGGCCCCCTTGCTTCATCTAGATC‐3′	11540–11564		
3'‐Inner	5′‐AAAGACGAGCCAGCGGTTAGTTGA‐3′	11002–11025	560	
3'RACE	5′‐CGCGGATCCTCCACTAGTGATTTCACACTATAGC‐3′	/		

### 2.3 Evolution and phylogenetic analysis of APPV genome

To determine the genetic characteristics of APPV, evolution and phylogenetic analysis was focused on the complete genome, N^pro^, E^rns^ and E2 genes of 58 APPV strains from different countries (including 6 strains from Guangxi province, China) available in GenBank on February 29, 2020 (Table 4). Nucleotide and amino acid identity analysis were calculated using MegAlign program of the DNAStar package (DNASTAR, USA). Phylogenetic trees were constructed by MEGA X (http://www.megasoftware.net/). Recombination evaluation was analysed using Recombination Detection Program 4 (RDP4) and then confirmed with SimPlot software (version 3.5.1). Bayesian inference analysis was performed using BEAST software package (version 1.10.4) (http://beast.community), and the times of the most recent common ancestor (tMRCA) and mean rate of molecular evolution were calculated by BEAST software.

## 3 RESULTS

### 3.1 Determination of APPV genome from positive samples

All the tissue samples collected from 53 piglets with CT were detected by PCR/RT‐PCR, and all of them were negative for CSFV, PCV2, PRV and JEV, whereas 41 of them were positive for APPV (41/53, 77.36%).

Five APPV‐positive samples were selected randomly for amplification and sequencing, and finally, the complete genome of five APPV strains were obtained. The complete genomes were 11 534–11 565 nucleotides (nt) in full‐length, with a 5′ UTR of 358–378 nt, followed by a single large ORF and a 3′ UTR of 268–279 nt. The ORF was 10 908 nt in length, which encoded a polyprotein of 3 635 amino acids (aa). The polyprotein was composed of 12 proteins, including four structural proteins (C, E^rns^, E1 and E2) and eight nonstructural proteins (N^pro^, P7, N2, NS3, NS4A, NS4B, NS5A and NS5B) (Table 2). Five APPV strains were named as GX04/2017, GX01‐2018, GX02‐2018, GX01‐2019 and GX02‐2019, respectively, and their GenBank accession numbers were MH102210, MH715893, MK453045, MN564752 and MN729215, respectively.

**Table 2:** The genome organization of APPV strains obtained in this study

Genome	Coded protein	Position	Nucleotide (nt)	Amino acid (aa)
5′ UTR	/	1–378	358–378	/
ORF	N^pro^	379–918	540	180
	C	919–1251	333	111
	E^rns^	1252–1881	630	210
	E1	1882–2478	597	199
	E2	2479–3201	723	241
	P7	3202–3393	192	64
	NS2	3394–4335	942	314
	NS3	4336–6396	2061	687
	NS4A	6397–6597	201	67
	NS4B	6598–7614	1,017	339
	NS5A	7615–9030	1,416	472
	NS5B	9031–11286	2,253	751
3′ UTR	/	11287–11564	278–279	/

Note:

The complete genomic sequence of GX02‐2018 (MK453045), GX01‐2019 (MN564752) and GX02‐2019 (MN729215) strains was 11 564 nt in full‐length, with a 5′ UTR of 378 nt, followed by an ORF of 10 908 nt and a 3′ UTR of 278 nt. The complete genomic sequence of GX04/2017 (MH102210) /GX01‐2018 (MH715893) was 11 534/11 565 nt in full‐length, with a 5′ UTR of 358/378 nt, followed by an ORF of 10 908 nt and a 3′ UTR of 268/279 nt.

### 3.2 Sequence analysis of APPV genome

Sequence analysis revealed that the nucleotide identities of the complete genome, ORF, N^pro^, E^rns^ and E2 genes among six APPV strains from Guangxi province were 83.3%‐97.5%, 83.0%‐98.2%, 79.6%‐97.2%, 80.8%‐98.4% and 83.4%‐96.8%, respectively; and the amino acid identities of ORF, N^pro^, E^rns^ and E2 genes were 91.7%‐99.1%, 81.1%‐97.8%, 89.5%‐99.0% and 90.5%‐96.3%, respectively (Table 3). The nucleotide identities of the complete genome, ORF, N^pro^, E^rns^ and E2 genes among six APPV strains from Guangxi province and the reference strains from different countries were 77.7%‐97.7%, 80.8%‐98.6%, 77.4%‐99.3%, 80.3%‐98.9% and 79.8%‐98.9%, respectively, whereas the amino acid identities of ORF, N^pro^, E^rns^ and E2 genes were 90.6%‐99.3%, 79.4%‐99.4%, 88.1%‐100% and 88.0%‐98.8%, respectively (Table 3).

**Table 3:** Nucleotide and amino acid identity (%) of identified strains and reference strains of APPV

Strain	Nucleotide and amino acid identity among identified strains					Nucleotide and amino acid identity between identified strains and reference strains				
	Genome	ORF	N^pro^	E^rns^	E2	Genome	ORF	N^pro^	E^rns^	E2
APPV_GX‐CH_2016	83.3–97.5	83.5–98.2 92.0–99.1	79.8–97.2 81.1–97.8	82.4–98.4 89.5–99.0	83.4–96.8 90.5–96.3	79.0–97.7	81.3–97.8 91.1–99.1	77.4–97.2 79.4–98.3	80.6–98.9 88.1–98.6	81.1–97.4 88.0–96.7
GX04/2017	83.3–88.0	83.0–87.9 91.7–94.8	79.6–87.8 81.1–89.4	80.8–86.7 90.5–94.3	84.9–89.1 91.3–94.2	78.1–97.2	81.0–98.5 90.7–99.1	78.1–98.7 80.0–98.9	80.3–98.4 91.0–99.5	79.8–98.9 88.8–98.3
GX01‐2018	84.1–97.5	83.6–98.2 92.1–99.1	79.8–97.2 81.7–97.8	82.5–98.4 89.5–99.0	83.5–96.8 91.7–96.3	78.2–97.7	81.3–98.6 91.1–99.3	77.4–99.3 80.0–99.4	80.3–98.6 88.1–99.5	80.9–98.1 89.2–97.9
GX02‐2018	84.2–89.7	83.6–89.4 92.3–95.2	80.7–90.4 81.7–91.1	82.4–88.6 92.4–94.3	86.4–91.6 92.5–95.4	78.3–89.7	81.4–89.7 91.4–96.0	78.0–91.7 79.4–93.3	80.6–89.4 91.0–95.7	82.3–92.0 90.5–96.7
GX01‐2019	84.0–90.9	83.5–90.7 91.9–95.4	83.0–90.4 83.9–91.1	83.0–90.2 90.0–93.8	84.9–91.6 92.9–95.4	78.3–97.6	81.5–98.1 91.1–99.2	78.3–98.1 80.6–97.2	81.4–98.4 90.0–100	80.8–98.3 90.9–98.8
GX02‐2019	83.3–84.2	83.0–83.6 91.7–92.3	79.6–83.0 81.1–83.9	80.8–83.0 89.5–92.4	83.4–86.4 90.5–93.8	77.7–95.9	80.8–96.9 90.6–98.6	78.1–97.8 80.0–96.7	80.8–96.5 88.1–98.1	80.6–97.8 88.4–98.3

### 3.3 Phylogenetic analysis of APPV genome

**Figure 1:**
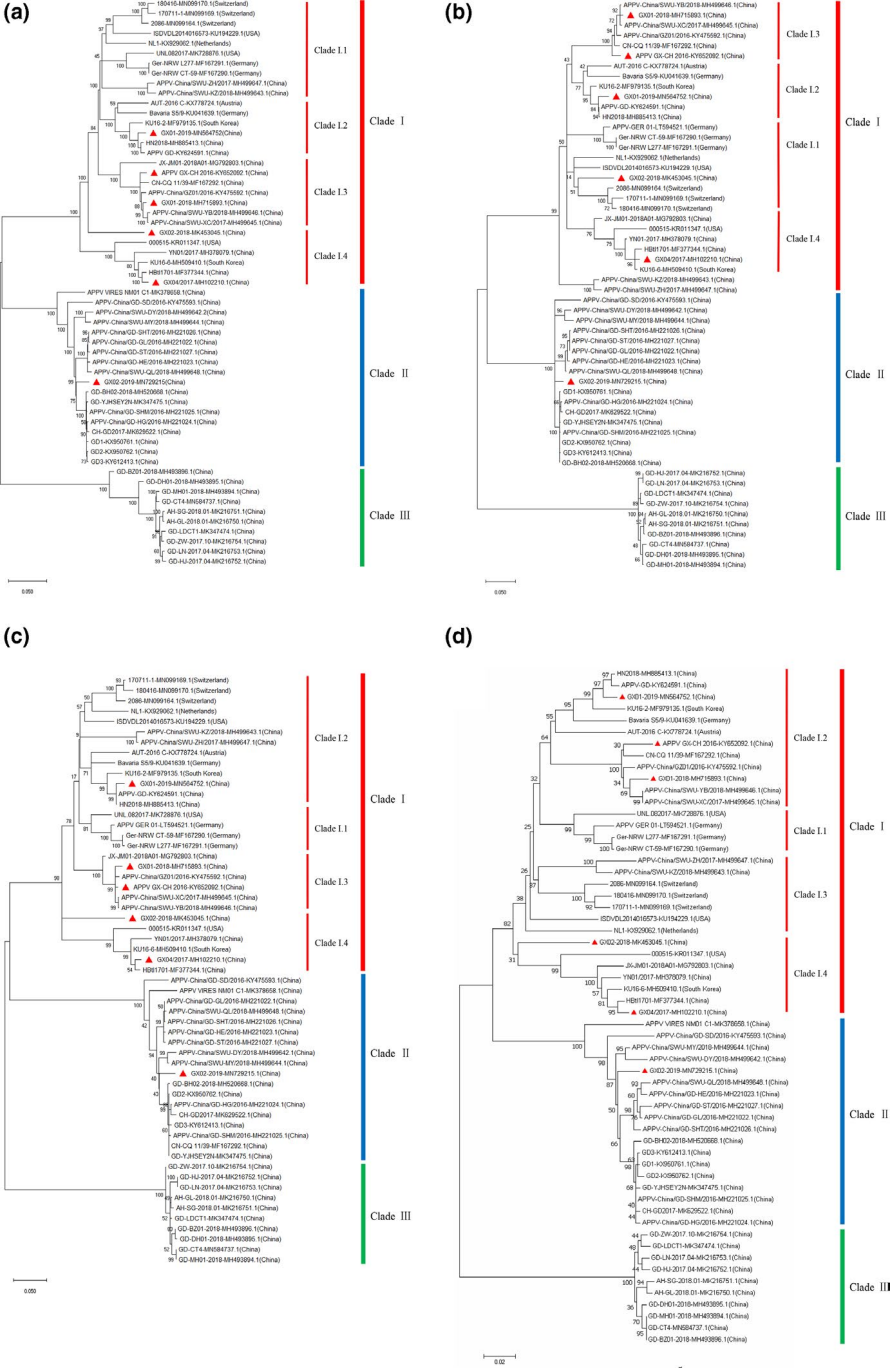
Phylogenetic trees based on the complete genome (a), Npro (b), Erns (c) and E2 (d) gene sequences of APPV strains from Guangxi province and other reference strains available in GenBank. Bootstrap values are shown at the nodes. Red solid triangle represents APPV strain from Guangxi province, China

Phylogenetic trees were constructed using the complete genome, N^pro^, E^rns^ and E2 gene sequences of 58 APPV strains available in GenBank (Table 4) and all APPV strains could be divided into three clades. The phylogenetic trees based on N^pro^, E^rns^ and E2 gene sequences showed quite similar topology with that of the complete genome (Figure 1). It was noteworthy that APPV_GX_Ch2016, GX04/2017, GX01‐2018, GX02‐2018 and GX01‐2019 strains from Guangxi province, together with 000,515 strain from the United States, KU16‐2/KU16‐6 strains from Korea, Bavaria S5/9 strain from Germany and AUT‐2016_C strain from Austria, belonged to clade I, but distributed in different subclades; GX02‐2019 strain, together with other Chinese strains, belonged to clade II; and all strains in clade III came from China.

**Table 4:** APPV reference strains used in this study

Strain	Accession number	Area	Collection date
2086	MN099164	Switzerland	2006
NL1	KX929062	The Netherlands	2012
000515	KR011347	USA	2014
ISDVDL2014016573	KU194229	USA	2014
CN‐CQ_11/39	MF167292	China	2014
Bavaria_S5/9	KU041639	Germany	2015
Ger‐NRW_CT‐59	MF167290	Germany	2015
AUT‐2016_C	KX778724	Austria	2016
APPV_GX‐CH_2016	KY652092	China	2016
KU16‐2	MF979135	Korea	2016
KU16‐6	MH509410	Korea	2016
GD1	KX950761	China	2016
GD2	KX950762	China	2016
GD3	KY612413	China	2016
APPV‐China/GZ01/2016	KY475592	China	2016
APPV‐China/GD‐SD/2016	KY475593	China	2016
APPV_GD	KY624591	China	2016
APPV‐China/GD‐GL/2016	MH221022	China	2016
APPV‐China/GD‐HE/2016	MH221023	China	2016
APPV‐China/GD‐HG/2016	MH221024	China	2016
APPV‐China/GD‐SHM/2016	MH221025	China	2016
APPV‐China/GD‐SHT/2016	MH221026	China	2016
APPV‐Ch**i**na/GD‐ST/2016	MH221027	China	2016
APPV_GER_01	LT594521	Germany	2016
Ger‐NRW_L277	MF167291	Germany	2016
UNL.082017	MK728876	USA	2017
170711–1	MN099169	Switzerland	2017
APPV_VIRES_NM01_C1	MK378658	China	2017
HBtl1701	MF377344	China	2017
GX04/2017	MH102210	China	2017
APPV‐China/SWU‐XC/2017	MH499645	China	2017
APPV‐China/SWU‐ZH/2017	MH499647	China	2017
CH‐GD2017	MK629522	China	2017
GD‐HJ‐2017.04	MK216752	China	2017
GD‐LN‐2017.04	MK216753	China	2017
GD‐YJHSEY2N	MK347475	China	2017
GD‐ZW‐2017.10	MK216754	China	2017
YN01/2017	MH378079	China	2017
JX‐JM01‐2018A01	MG792803	China	2018
APPV‐China/SWU‐DY/2018	MH499642	China	2018
APPV‐China/SWU‐KZ/2018	MH499643	China	2018
APPV‐China/SWU‐MY/2018	MH499644	China	2018
APPV‐China/SWU‐QL/2018	MH499648	China	2018
APPV‐China/SWU‐YB/2018	MH499646	China	2018
GD‐BH02‐2018	MH520668	China	2018
GD‐BZ01‐2018	MH493896	China	2018
GD‐LDCT1	MK347474	China	2018
GD‐CT4	MN584737	China	2018
GD‐DH01‐2018	MH493895	China	2018
GD‐MH01‐2018	MH493894	China	2018
180416	MN099170	Switzerland	2018
AH‐GL‐2018.01	MK216750	China	2018
AH‐SG‐2018.01	MK216751	China	2018
HN2018	MH885413	China	2018
GX01‐2018	MH715893	China	2018
GX02‐2018	MK453045	China	2018
GX01‐2019	MN564752	China	2019
GX02‐2019	MN729215	China	2019

### 3.4 Evolution analysis of APPV strains

**Figure 2:**
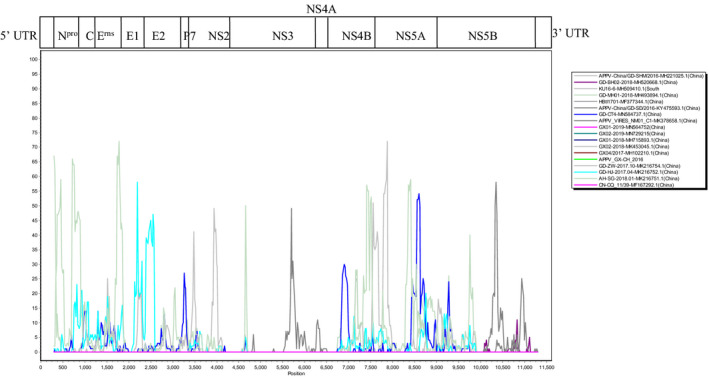
Recombination analysis on the complete genomes of six APPV strains from Guangxi province. Potential recombination events were identified using Recombination Detection Program 4 (RDP4) and then examined using similarity plots and bootstrap analysis Simplot 3.5.1. The major and minor parents were represented in Table 5

Recombination Detection Program 4 (RDP4) and Simplot 3.5.1 were used to analyse the recombination events of 58 APPV strains from different countries available in GenBank (Table 4). The results showed that there existed recombination detection signs in six strains, including GD‐BZ01‐2018 (MH493896), GD‐DH01‐2018 (MH493895), YN01/2017 (MH378079), JX‐JM01‐2018A01 (MG792803), GD‐LN‐2017.04 (MK216753) and GD2 (KX950763) (Table 5), and all of them came from China. However, no sign of recombination was detected in all six strains from Guangxi province (Figure 2).

**Table 5:** Recombination analysis of APPV complete genomes

Strain	Major Parent/Similarity (%)	Minor Parent/Similarity (%)	Breakpoint position in alignment
GD‐BZ01‐2018	AH‐SG‐2018/98.8	GD‐CT4/100	2138‐2537/3253‐3678
	GD‐MH01‐2018/98.6	APPV_VIRES_NM01_C1/97.9	4712‐4782/6590‐6618
	GD‐CT4/95.7	GD2/93.4	9950‐9984/11400‐361
GD‐DH01‐2018	GD‐CT4/99.4	GD2/99.4	9834–9868
YN01/2017	KU16‐6/97.2	APPV‐China/GD‐SD/2016/97.2	6453–6498
JX‐JM01‐2018A01	HBtl1701/97.3	APPV_GX‐CH_2016/95.8	10895‐11004/220‐570
	HBtl1701/95	CN‐CQ_11/39/97.1	845‐1113/2240‐2325
	GX01‐2018/93.7	APPV_VIRES_NM01_C1/97.3	3127‐3294/3809‐3870
	HBtl1701/96.2	CN‐CQ_11/39/97.7	4196‐4618/5784‐5937
	GX04/2017/93.5	GX01‐2018/98.6	6236‐6342/9095‐9198
GD‐LN‐2017.04	GD‐ZW‐2017.04/99.3	GD‐HJ‐2017.04/99.9	9352‐11053/2118‐2771
GD2	APPV‐China/GD‐SHM/2016/99.7	GD‐BH02‐2018/100	5084‐5602/6152‐8469

Evolutionary estimation based on the complete genome, N^pro^, E^rns^ and E2 gene sequences of 58 APPV strains from different countries was conducted by Bayesian analysis. The results indicated that APPV genomic sequences evolved at a mean rate of 1.37 × 10^–4^ (95% highest probability density, HPD: 5.12 × 10^–6^–3.02 × 10^–4^) substitutions/site/year (s/s/y), and the mean rates of molecular evolution of N^pro^, E^rns^ and E2 gene sequences were 1.00 × 10^–4^ (8.47 × 10^–5^–1.17 × 10^–4^), 1.56 × 10^–4^ (9.81 × 10^–5^–2.20 × 10^–4^) and 1.01 × 10^–4^ (8.17 × 10^–5^–1.22 × 10^–4^) s/s/y, respectively. Bayesian inferences (BI) estimated that the tMRCA of complete genomes of APPV strains existed 1,700.5 (95% HPD: 228.4–4654.5) years ago, and the tMRCAs of N^pro^, E^rns^ and E2 gene sequences were 1,495.5 (1,404.3–1597.4), 1,390.8 (821.9–2032.2) and 1823.0 (1723.2–1918.1) years ago, respectively.

## 4 DISCUSSION

CT has been classified as type A and type B, of which type A is associated with variable hypomyelination of brain and spinal cord, whereas histopathological lesions are missing in type B (de Groof et al., 2016). Type A‐Ⅱ in newborn piglets was characterized by generalized shaking and varying degrees of absence of myelin sheath in brain and spinal cord (Mosena et al., 2018). It was first reported in 1922, but the causative agent has always been a mystery until APPV was identified in the United States in 2015 and then in other countries (Arruda et al., 2016; Denes et al., 2018; Dessureault et al., 2018; Gatto et al., 2018; Kaufmann et al., 2019; Mosena et al., 2018; Munoz‐Gonzalez et al., 2017; Postel et al., 2016; Schwarz et al., 2017; Sozzi et al., 2019; Williamson, 2017; Zhang et al., 2017). APPV have been detected in serum, thymus, peripheral lymphoid organs (spleen, tonsil, submaxillary lymph node and inguinal lymph node), nervous system (brain stem, brain and cerebellum), digestive system (duodenum) and semen (Gatto et al., 2018; Hause et al., 2015; Munoz‐Gonzalez et al., 2017; Postel et al., 2017; Yuan et al., 2017), which indicated that this virus has widespread tissue tropism. However, it was very difficult for this virus to culture and identify. Many researchers have attempted to seek appropriate cell lines to culture and acquire a higher titre of APPV for identification and characterization, but unfortunately, no one has been successful until now because this virus could not replicate in the selected cell lines or the viral titre was too low (Cagatay et al., 2018; Gatto et al., 2018; de Groof et al., 2016; Hause et al., 2015; Postel et al., 2016; Schwarz et al., 2017). Until now, all the APPV sequences obtained were directly amplified, cloned and sequenced from positive serum and tissue samples (de Groof et al., 2016; Hause et al., 2015; Pan, Yan, et al., 2019; Schwarz et al., 2017; Shen et al., 2018; Zhang et al., 2018). Therefore, we also amplified and sequenced the APPV genome of all five Guangxi strains from clinical tissue samples and used to analyse their evolution and genetic diversity in this study.

Sequence analysis revealed that six APPV strains from Guangxi province shared 83.3%‐97.5% nucleotide identity with each another, and shared 77.7%‐97.7% nucleotide identity with other reference strains, showing that the most genetic distance reached 22.3%. Similar results have been reported by other studies. The ORF sequences of German APPV were demonstrated to be highly variable from different domestic regions with genetic variability of 81%‐87% among different isolates (Beer et al., 2017; Postel et al., 2016). The genomic sequences of Austrian APPV shared 90% nucleotide identity with those from the United States and 92% nucleotide identity with those from Germany (Schwarz et al., 2017). The APPV strains from Guangdong province in China shared 80.5%‐84.1% nucleotide identity with other APPV strains from different countries (Zhang et al., 2019). These results indicated that there existed a high genetic diversity of current APPV strains from different countries around the world.

Phylogenetic analysis revealed that all 58 APPV strains from different countries could be divided into three clades, and the six APPV strains from Guangxi province distributed in two clades (clade I and II). Clade I strains came from America, Europe and Asia, whereas clade II and III strains came from China. Five strains from Guangxi province (APPV_CH‐GX_2016, GX04/2017, GX01‐2018, GX02‐2018 and GX01‐2019) distributed in clade I, and one strains from Guangxi province (GX02‐2019) distributed in clade II. One study reported that 12 APPV strains from Guangdong province distributed in three well‐defined clades, which showed high genetic diversity among these strains (Yan et al., 2019). The results suggested that not only within a country but also within an area, such as Guangxi province, APPV exhibited a high genetic diversity among different strains.

Recombination analysis of 58 APPV strains showed that no recombination in all six strains from Guangxi province, and all the six recombinant strains came from other provinces in China. An identical result was confirmed by the SimPlot software analysis. Similarity analysis of APPV genomes indicated that there existed high variation regions in whole genomic sequences, which would be a big challenge for molecular diagnosis and epidemiological investigation of APPV. It was noteworthy that the similarity relationship of GX01‐2018 strain was close to NL1 and AUT‐2016_C strains from Europe, whereas GX04/2017 strain presented relative higher similarity to 000,515 strain from North America, suggesting again that there existed high genetic diversity and variation of APPV strains from Guangxi province.

To our knowledge, this is the first report on the estimation of the mean rate of molecular evolution and dates of the tMRCA about APPV. The results revealed that APPV complete genome, N^pro^, E^rns^ and E2 gene evolved at an evolutionary rate of 1.37 × 10^–4^ 1.00 × 10^–4^, 1.56 × 10^–4^ and 1.01 × 10^–4^ s/s/y, respectively, and their tMRCA were estimated as 1,700.5, 1,495.5, 1,390.8 and 1823.0 years ago, respectively, which indicated that N^pro^, E^rns^ and E2 genes had similar evolutionary rate and tMRCA with the complete genome. Some previous studies had been done on the evolution of other pestiviruses. A mean substitution rate of 1.4 × 10^–3^ s/s/y was found across both bovine viral diarrhoea virus 1 (BVDV‐1) and BVDV‐2 and the tMRCA was estimated to be 1,498 years ago (Chernick & Meer, 2017); the mean evolutionary rate of the 5' UTR sequences of border disease virus (BDV) was 2.9 × 10^−3^ s/s/y (Luzzago et al., 2016). The full‐length E2 gene of CSFV from different countries had an evolutionary rate of 3.2 × 10^–4^ s/s/y and the origin of CSFV was estimated to be the mid‐1500s (Garrido Haro et al., 2018), whereas the whole CSFV genome evolved at a rate of 1.03 × 10^–4^ s/s/y and the tMRCA appeared 2,770.2 years ago (Kwon et al., 2015). As for other highly variable RNA virus, such as porcine reproductive and respiratory syndrome virus (PRRSV) and swine influenza virus (SIV), some studies reported that the evolutionary rate of PRRSV genome ranged from 1.98 × 10^–3^ to 3.29 × 10^–3^ s/s/y (Song et al., 2010; Yoon et al., 2013) and that of SIV HA gene ranged from 1.03 × 10^–3^ to 3.18 × 10^–3^ s/s/y (Al Khatib et al., 2018; Wei et al., 2016). These results showed that the evolutionary rate of APPV was similar to that of CSFV, and lower than those of other RNA viruses. This study might provide a complementary reference for evolutionary information of APPV.

In summary, the complete genomic sequences of five APPV strains were obtained from positive clinical tissue samples from neonatal piglets with CT in Guangxi province, Southern China. The five APPV strains distributed in two clades of phylogenetic trees based on complete genome, N^pro^, E^rns^ and E2 gene sequences, respectively, which indicated that APPV strains from Guangxi province existed high degree of genetic diversity and variation. The results provided valuable information on the epidemiological and evolutionary characteristics of APPV in Southern China.

## CONFLICT OF INTEREST

The authors declared no potential conflict of interest with respect to the research, authorship and publication of this article.

## AUTHOR CONTRIBUTIONS

**Kaichuang Shi:** Project administration; Supervision; Writing‐review & editing. **Shouyu Xie:** Investigation; Writing‐original draft. **Hui‐xin Liu:** Investigation. **Jing Zhao:** Software. **Sujie Qu:** Investigation; Methodology. **Wenjun Lu:** Investigation.

### Peer Review

The peer review history for this article is available at https://publons.com/publon/10.1002/vms3.407.
